# Supplementary material for: Satisfaction and continuance intention of blended learning from perspective of junior high school students in the directly-entering-socialism ethnic communities of China
Source: PLoS One. 2022 Dec 1;17(12):e0270939. doi: 10.1371/journal.pone.0270939 (PMC9714753; doi:10.1371/journal.pone.0270939)
Supplement: Attachment — Submitted filename: Response to reviewers.docx [file pone.0270939.s001.docx]

Rebuttal Letter to manuscript PONE-D-22-16934:

“Satisfaction and continuance intention of blended learning from perspective of junior high school students in the directly-entering-socialism ethnic communities of China”

Dear Editor, and Dear Reviewers,

We would like to thank you for your comments and suggestions, which gave us a lot of help to improve the quality of manuscript. In the revised manuscript, we revised all the issues raised. In this document, we answered all the questions proposed by journal, editor and the reviewers. Comments are shown in black bold font, followed by our answer/comment in blue normal font. The major corrections/changes in the manuscript are displayed in red font and blue front.

**Journal Requirements:**

1. **Please ensure that your** **manuscript meets PLOS ONE's style requirements, including those for file naming.**

Answer:

Yes, we have made manuscript meet PLOS ONE's style requirements, including those for file naming. What’s more, pages and line numbers were added. Abstract was modified to 288 words(not exceed 300 words).

2. **PLOS ONE does not copy edit accepted** **manuscripts. To that effect, please ensure that your submission is free of typos and grammatical errors.**

Answer:

Yes, we have checked the words and grammar ensuring the manuscript is free of typos and grammatical errors.

3. **We note that you have stated that you will provide repository information for your data at acceptance. Should your manuscript be accepted for publication, we will hold it until you provide the relevant accession numbers or DOIs necessary to access your data. If** **you wish to make changes to your Data Availability statement, please describe these changes in your cover letter and we will update your Data Availability statement to reflect the information you provide.**

Answer:

For data availability statement, we provided repository information for our data at acceptance in this letter. The relevant DOI is 10.57760/sciencedb.03107. The private access link is https://www.scidb.cn/s/iMRZ7r.

4. **Please amend either the abstract** **on the online submission form (via Edit Submission) or the abstract in the manuscript so that** **they are identical.**

Answer:

We have amended abstract both identical.

5. **We note you have included a table to which you do not refer in the text of your manuscript. Please ensure that you refer to Table 7 in your text; if accepted, production will need this reference to link the reader to the Table.**

Answer:

We have amended the mistakes in the text. You can check it on line numbers 312 in revised manuscript with track changes.

6. **Please review your** **reference list to ensure that it is complete and correct. If you have cited papers that have been retracted, please include the rationale for doing so in the manuscript text, or remove these references and replace them with relevant current references. Any changes to the reference list should be mentioned in the rebuttal letter that accompanies your revised manuscript. If you need to cite a retracted article, indicate the article’s retracted status in the References list and also include a citation and full reference for the retraction notice.**

Answer:

We have amended reference format as outlined in the ICMJE sample references as PLOS ONE requires. And we added the updated references in order as reviewers comments. Details was described as follow:

For introduction part, we added four references from 6 to 9 in reference list (line number 95-99 in the text).

For table1, we added fourteen references from 30 to 43 in reference list (line number 202-208 in the text).

For discussion part, we added four references from 68 to 71 in reference list (line number 383-386 in the text).

**Additional Editor Comments:
The authors have proposed a blended learning process from perspective of junior high school students in the directly-entering-socialism ethnic communities of China. The reviewer appreciate the authors efforts. However, following comments need to be addressed:**

**1. In Section Previous research and research model, what is "[Error! Reference source not found.]"**

Answer:

We have amended this mistake on line number 149. It was a wrong hyperlink which displayed error in PDF file.

**2. The label of "Figure 2. Result of the Structural Model" is not properly placed.**

Answer:

We have amended this mistake on line number 333.

**3. In table 1,** **more recent papers need to be added.**

Answer:

We have added more recent papers.

Perceived Usefulness: Almaiah et al. (2019), Alkhawaja et al. (2022).

Satisfaction: Almaiah et al. (2019), Warren et al. (2020)

Confirmation: Yang et al. (2022)

Self-efficacy: Vermunt& Donche (2017), Yang et al. (2022)

Information Quality: Almaiah et al. (2019), Alfaki et al. (2021)

Continuance Intention: Li et al. (2021), Yang et al. (2022)

**Review Comments:**

**Reviewer #1:**

**Introduction section is very short, Motivation of the work should be added in the Introduction and also challenges in the current system.**

**proposed methodology could be represent in the scientific manner.**

**Its good authors have provided the sufficient results however the representation of the manuscript need to be done.**

**Latest work should be added, Update the** **conclusion & abstract accordingly.**

**Rashid, E., Ansari, M. D., Gunjan, V. K., & Khan, M. (2020). Enhancement in teaching quality methodology by predicting attendance using machine learning technique. In Modern approaches in machine learning and cognitive science: a walkthrough (pp. 227-235). Springer, Cham.**

**Sethi, K., Jaiswal, V., & Ansari, M. D. (2020). Machine learning based support system for students to select stream (subject). Recent advances in computer science and communications (Formerly: Recent patents on computer science), 13(3), 336-344.**

**Rashid, E., Ansari, M., & Gunjan, V. K. (2022). Innovation and Entrepreneurship in the Technical Education. In ICCCE 2021 (pp. 1199-1203). Springer, Singapore.**

Answer:

We thank you for the general appreciation of our work, and specific comments and references given that help to improve our manuscript.

We added the motivation of the work and challenges in the current system in the Introduction as mentioned. For the result presentation, we divided it into four parts, i.e. demographic information, reliability and validity, model fit, and hypothesis testing. For each part, we described the data of the result following a figure. And Proposed methodology was modified.

We thank you for giving us the three references to review. It inspires us a lot for the further study. We read more articles and books on machine learning. We updated and added these references in the Discussion considering that the conclusion and abstract should keep objectively described focused on the research objectives after intense discussion and deep thinking. In the discussion, we think the research model will hopefully be used in machine learning to predict student satisfaction and continuance intention of blended learning with experience data.

**Reviewer #2:**

**The authors have proposed a blended learning process from perspective of junior high school students in the directly-entering-socialism ethnic communities of China. The reviewer appreicate the authors efforts. However, following comments need to be addressed:**

**1. In Section Previous research and research model, what is "[Error! Reference source not found.]"**

**2.** **The label of "Figure 2. Result of the Structural Model" is not properly placed.**

**3. In table 1, more recent papers need to be added.**

Answer:

We thank you for recognition of our efforts, and specific comments.

We Revised accordingly.

For "[Error! Reference source not found.]", We have amended this mistake on line number 149. It was a wrong hyperlink which displayed error in PDF file.

For the label of "Figure 2", we replaced as the journal requires.

For table 1, we have added more recent papers.

Perceived Usefulness: Almaiah et al. (2019), Alkhawaja et al. (2022).

Satisfaction: Almaiah et al. (2019), Warren et al. (2020)

Confirmation: Yang et al. (2022)

Self-efficacy: Vermunt& Donche (2017), Yang et al. (2022)

Information Quality: Almaiah et al. (2019), Alfaki et al. (2021)

Continuance Intention: Li et al. (2021), Yang et al. (2022)

**Reviewer #3:**

**Paper is based on survey.**

**A satisfactory amount of data has been collected and analyzed.**

**Results are well organized and calculated.**

**Conclusion parts meets the objective of the research statement.**

**References are sufficient and latest.**

Answer:

We thank you for the specific comments of our manuscript.

Beast regards

Chunyu li
